# State-of-the-Art Review of Electrospun Gelatin-Based Nanofiber Dressings for Wound Healing Applications

**DOI:** 10.3390/nano12050784

**Published:** 2022-02-25

**Authors:** Tao Li, Mingchao Sun, Shaohua Wu

**Affiliations:** College of Textiles & Clothing, Qingdao University, Qingdao 266071, China; tiaoli2022@163.com (T.L.); 15662505526@163.com (M.S.)

**Keywords:** electrospinning, gelatin, nanofiber, wound dressing, skin regeneration

## Abstract

Electrospun nanofiber materials have been considered as advanced dressing candidates in the perspective of wound healing and skin regeneration, originated from their high porosity and permeability to air and moisture, effective barrier performance of external pathogens, and fantastic extracellular matrix (ECM) fibril mimicking property. Gelatin is one of the most important natural biomaterials for the design and construction of electrospun nanofiber-based dressings, due to its excellent biocompatibility and biodegradability, and great exudate-absorbing capacity. Various crosslinking approaches including physical, chemical, and biological methods have been introduced to improve the mechanical stability of electrospun gelatin-based nanofiber mats. Some innovative electrospinning strategies, including blend electrospinning, emulsion electrospinning, and coaxial electrospinning, have been explored to improve the mechanical, physicochemical, and biological properties of gelatin-based nanofiber mats. Moreover, numerous bioactive components and therapeutic agents have been utilized to impart the electrospun gelatin-based nanofiber dressing materials with multiple functions, such as antimicrobial, anti-inflammation, antioxidation, hemostatic, and vascularization, as well as other healing-promoting capacities. Noticeably, electrospun gelatin-based nanofiber mats integrated with specific functions have been fabricated to treat some hard-healing wound types containing burn and diabetic wounds. This work provides a detailed review of electrospun gelatin-based nanofiber dressing materials without or with therapeutic agents for wound healing and skin regeneration applications.

## 1. Introduction

Skin is the largest organ located on the surface of human body, providing some essential sensing, thermoregulation, biological and immune functions, as well as continuous physical protection against various external pathogens [1,2]. Due to the long-term and continuous exposure to both external and internal environment, some skin damages occur inevitably. Specifically, various injuries originated from mechanical, thermal, and chemical actions, and various ulcers from chronic pathophysiological conditions can cause different degrees of skin wounds [3,4]. It has been reported that a wound with a size more than 10% of the whole human skin area is life-threatening because of the large loss of extracellular fluid [5].

Wound dressings are one essential type of medical device to cover the open wound for protect the wound against the external risk factors while providing an appropriate microenvironment to support the wound healing process [6,7]. The use of dressing material to cover the skin wound dates to 1880 when a sandwiched dressing made of cotton and wool was employed to process a skin wound by Joseph Gamgee [8]. Comparing with some conventional wound dressing materials, such as gauzes, tulles, bandages, the design and development of some innovative multifunctional wound dressings including hydrocolloids, hydrogels, and nanomaterials are urgently required [9,10,11]. The multifunctional properties referred to here, include antimicrobial, hemostatic, vascularization, and immune regulation, as well as other healing-promoting capacities, except for the traditional physical protection and exudate-absorbing functions [12,13,14]. The advances of novel wound dressing materials are expected to shorten the healing period and improve the functions of as-healed wound.

Most recently, electrospinning has been widely explored as a promising technique to fabricate nanofiber nonwoven mats, which have attracted intense attention in the fields of regenerative medicine and tissue engineering, such as wound healing, drug carrier, as well as osteochondral and gastrointestinal tissue engineering, etc. [15]. As wound dressing materials, electrospun nanofiber mats possess several outstanding merits. Firstly, the decreased fiber scale of electrospun nanofibers can endow the dressing materials with an obviously enhanced specific surface area comparing with the microfibers made from traditional melt, dry or wet spinning, thus providing more cell adhesion and growth sites [16,17]. The nanofibers generated by electrospinning can also better replicate the dimension and structure of protein fibrils existed in the extracellular matrix (ECM) of native skin organ, inevitably producing a healing-promoting microenvironment for wound healing and skin regeneration [18,19]. Secondly, the electrospun nanofibers possess a nonwoven mat-like structure with small pore size and high porosity, which can effectively block the invasion of external pathogens while provide great air and moisture permeability [20,21]. Thirdly, electrospinning technique can also be utilized as drug carriers to load various bioactive and therapeutic agents to improve the wound healing efficiency and regeneration outcomes [22,23].

Although there are more than one hundred different types of polymers electrospun into nanofibers, gelatin is assuredly one of the most important biopolymers for the fabrication of electrospun nanofiber mats for wound healing applications. Gelatin has excellent biodegradability and biocompatibility, good cell adhesion properties, and high possibility for modification and functionalization [24]. Moreover, gelatin generated from collagen has native ECM-like physicochemical properties, and outstanding wound exudate-absorbing capacity [25]. However, gelatin alone commonly exhibits poor electrospinnability and generates electrospun nanofibers with low quality due to its strong hydrogen bonding [26]. For example, the mechanical properties and structural stability of electrospun gelatin nanofibers are poor, and the fast in vivo solubility is also a fatal drawback for pure gelatin nanofibers [27]. Gelatin blending with some other natural or synthetic biopolymers can improve the spinnability and enhance the mechanical, physicochemical, and biological properties of generated electrospun nanofibers, which are more attractive for wound healing application [28,29]. Polymer modification and crosslinking are both important strategies for increasing the wet resistance and structural stability, as well as various performances of electrospun gelatin-based nanofiber mats [30,31,32].

**Figure 1:**
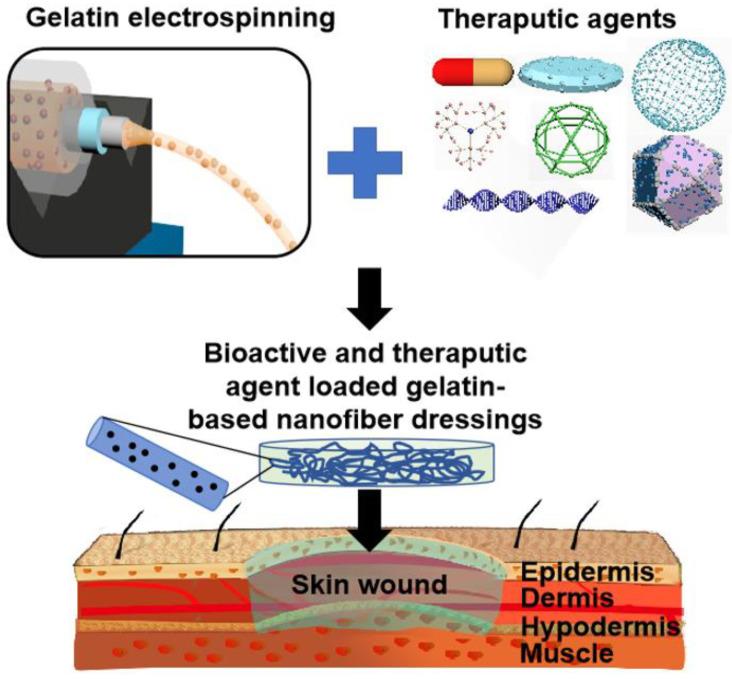
Schematic of the design and development of bioactive and therapeutic agent loaded gelatin-based nanofiber mats for wound treatment applications.

This review firstly introduces the common wound type, normal wound healing process, and general properties of gelatin polymer. Then the process and mechanisms of electrospinning as well as the representative morphology and structure of electrospun nanofibers are introduced. After that, this review summarizes the preparation of electrospinning gelatin-based nanofiber mats and their applications in wound healing and skin regeneration. Importantly, some innovative strategies like loading various bioactive and therapeutic agents including natural drugs, synthesized drugs, growth factors, and other biomolecules into electrospun gelatin-based nanofibers (Figure 1) are also highlighted. Eventually, the challenges and prospects of electrospinning gelatin-based dressings for clinical applications of wound treatment are deeply discussed.

## 2. Wound Type and Wound Healing Process

The Wound Healing Society (WHS) defines a wound as the injury or interruption of normal skin tissue structure accompanied with impaired skin functions [33]. According to the wound duration, the wounds can be categorized into acute wounds and chronic wounds [34,35]. The acute wounds with small areas of damaged skin can be healed spontaneously in a well-regulated manner. However, the large-scale acute wounds originated from burns or accidents fail to be self-healing [36]. Moreover, the chronic wounds caused by diabetic foot ulcers, pressure ulcers, arterial insufficiency ulcers, and venous ulcers also exhibit a pathologically delayed healing process, with the lesion remaining open and unhealed for more than three months [35,37]. Worse still, the long-term exposure of large-scale acute wounds or chronic wounds improves the possibility of various microorganism contamination and biofilm formation, leading to a local inflammation and infection, and even a systemic infection such as septicemia [38,39].

**Figure 2:**
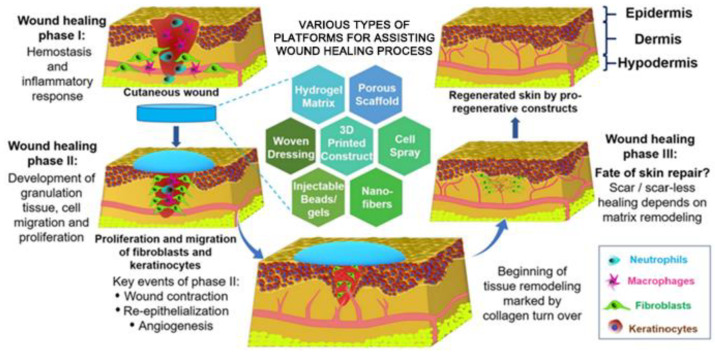
Summarization of some representative innovative platforms and dressing materials and Schematic of the representative concept and mechanisms of a wound healing process using an advanced dressing material. Reprinted with permission from ref. [45], Elsevier, 2019.

The normal healing process for skin wounds commonly refers to four phases, i.e., hemostasis, inflammation, proliferation, and ECM remodeling, which may overlap in space and time [40,41,42]. However, the healing process for large-scale acute wounds and chronic wounds may be held back at any of the above-referred four stages. Although they differ in the causes and pathological mechanisms, but commonly have some similar characteristics including extended inflammatory stage, decreased growth factor activity, and high protease activity, and poor vascularization, inevitably resulting in a prolonged healing process and inferior healing results [11,43,44]. Therefore, it is necessary to take appropriate measures for treating these hard-healing wounds in clinics. Wound dressings are one of most widely employed medical strategies to help the healing process of hard-healing wounds. The advances of science and technology have produced some innovative platforms and dressing materials, mainly including the hydrogel matrix, the porous scaffold, a woven dressing, a 3D printed construct, and nanofiber dressings [45]. Figure 2 shows a schematic illustration for the mechanisms of a wound healing process assisting with a dressing material [45]. The component, morphology, structure, and performances (especially biological properties) of the wound dressing materials are responsible for the regeneration of functional and scarless skin tissues or the formation of non-functional or semi-functional scar tissues.

## 3. Electrospinning Technique

**Figure 3:**
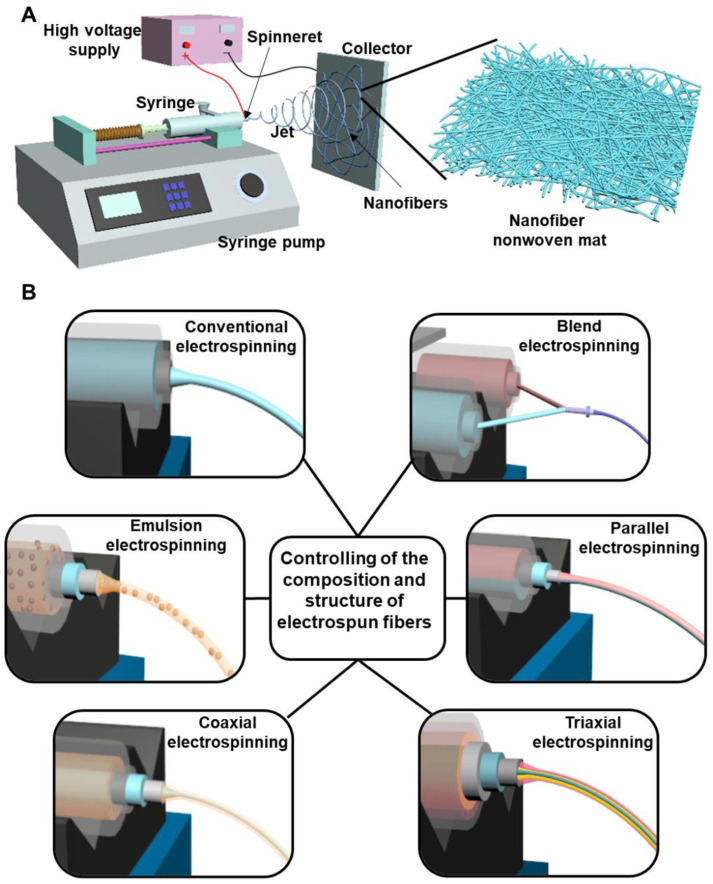
(**A**) Schematic of a conventional electrospinning device. (**B**) Schematic of some representative modified electrospinning strategies to control the composition and structure of electrospun fibers.

Electrospinning is a simple, versatile, and cost-effective strategy to generate polymeric fibers [46,47]. The diameters of electrospun fibers are in the range of several to several hundred nanometers, which are more than 100 times smaller than the fibers manufactured by traditional melt, dry or wet spinning approaches, thus have attracted intense interests as biomedical materials for wound healing applications [48,49]. The conventional electrospinning device contains several main components, i.e., a spinneret (commonly in the form of a blunt tip needle) connected with a syringe, a syringe pump, a high-voltage supply, and a collector (commonly in the form of a metal plate) (Figure 3A). The mechanisms and process of conventional electrospinning technique are summarized as following [50,51]. The spinning polymer solution is loaded into a syringe that is connected to a syringe pump, controlling the polymer solution with a predetermined supplying rate to the spinneret. A high-voltage supply is connected to the spinneret, and a high voltage generated by the high-voltage supply is the main driving form to produce an electrically charged polymeric jet from the polymer solution in the spinneret. Specifically, when a high voltage is applied to the spinneret, the electrostatic charges accumulate in a polymer droplet at the tip of spinneret, which gradually drives the change of droplet into a conical shape named as a Taylor cone. With the increasing of applied voltage, the repulsive electrostatic force originated from accumulated electrostatic charges overcomes the surface tension of polymer solution, and then an electrically charged polymeric jet is ejected from the tip of Taylor cone. The polymeric jet travels towards the grounded collector under the function of an electrical field generated between the spinneret and collector. The polymeric jet stretches in a straight line in the initial stage, and then experiences a bending and whipping stage. With the solvent volatilization, the stretched and solidified polymeric jet is deposited on the collector, and the nanofibers in the form of nonwoven mat with high porosity, small pore size, and ECM-mimicking structure are finally generated.

In conventional electrospinning, nanofibers with a single component and cylinder-like structure are produced [52,53]. To control and adjust the fiber component and structure, some modified electrospinning strategies have been designed and implemented. As shown in Figure 3B, they mainly contain blend electrospinning, emulsion electrospinning, coaxial electrospinning, parallel electrospinning, and triaxial electrospinning. Blend electrospinning is recognized as one of simplest method to fabricate blend nanofibers made from two or multiple different polymers [54]. The different polymers can be dissolved in the same solvent system for full mixing or can be dissolved separately and then on-line blended before electrospinning. Blend electrospinning has been employed to carry the therapeutic drugs with predetermined biological functions for biomedical applications [23]. If the polymer-used solvent cannot dissolve the drugs, a small amount of drug-dissolved solvent is firstly utilized to dissolve drug, and further added into the polymer solution for blend electrospinning. Emulsion electrospinning is defined as the electrospinning of polymer emulsion, which is also widely used to effectively load various types of drugs [55,56]. For the hydrophilic drugs, they can be dissolved into water and then diffused into a polymer dissolved oil phase solvent with the help of an emulsifier for electrospinning, whereas for the hydrophobic drugs, they can be dissolved into oil solvent and then diffused into a polymer/water solution by an emulsifier for electrospinning. For those drugs having low dissolvability in the spinning solution, the emulsion electrospinning-based nanofibers exhibit more uniform drug distribution compared to drug-loaded fibers made from blend electrospinning [57]. Coaxial electrospinning is conducted with a coaxial spinneret to generate core-sheath structured nanofibers [58]. The core component and sheath component could be widely adjusted by using two spinning syringes. Moreover, loading drugs into the core layer by coaxial electrospinning can decrease the burst release and extend release time of loaded drugs compared with both blend electrospinning and emulsion electrospinning [59,60]. Based on modifying the coaxial electrospinning process, parallel electrospinning and triaxial electrospinning are designed. In parallel electrospinning, Janus nanofibers can be generated with a side-by-side structured spinneret [61,62]. In triaxial electrospinning, a triaxial spinneret made from three concentrically arranged needles are utilized to generate electrospun nanofibers with a triple-layered core-middle-shell structure [63,64]. If the drugs are loaded into the inner core layer, which can further decrease the initial burst and exhibit a prolonged release behavior, compared to the coaxial electrospinning-based fiber structure. Importantly, multiple different drugs and polymer components can be applied into the three different layers of triaxial electrospinning generated nanofibers by using three separate syringes [65].

## 4. Electrospun Gelatin-Based Nanofibers

### 4.1. Electrospun Nanofibers from Pure Gelatin

Electrospinning of gelatin provides a promising strategy to integrate the great performances of gelatin with the ideal morphology and structure of electrospun nanofibers. As a water-soluble polymer, water is used as a solvent to dissolve gelatin for electrospinning application. Unfortunately, the gelatin/water solution exhibited poor electrospinnability [66]. Firstly, the gelation process occurs when the temperature of gelatin/water solution is below 30 °C, so it is impossible to use electrospinning gelatin/water solution at room temperature. Secondly, the strong hydrogen bonds formed in gelatin molecules and between gelatin molecules and water molecules also hold back the initialization, formation, and stability of polymeric jets during the electrospinning process [26].

To improve the spinnability of gelatin/water solution, some acidic aqueous solvents have been widely explored to dissolve gelatin. The acidic solvent system can break the intermolecular crosslinks in gelatin and make the gelatin macromolecules exhibit random coil conformation and appropriate viscosity, which are suitable for electrospinning technique. Ki et al. dissolved gelatin into 98% (*v*/*v*) formic acid aqueous solution and demonstrated a good electrospinnability [67]. The as-electrospun gelatin nanofibers showed bead-free morphology with the diameters ranging from 70–170 nm. Okutan et al. employed an 20% (*v*/*v*) acetic acid aqueous solution for gelatin electrospinning and found that the as-generated gelatin nanofibers exhibited smooth surface morphology and thin diameters (several tens of nanometers) [68]. Songchotikunpan et al. used two different solvents, i.e., 80% (*v*/*v*) formic acid 40% (*v*/*v*) acetic acid to dissolve and electrospin gelatin and demonstrated that the diameters of obtained fibers ranged from 109 nm and 761 nm regardless of the acidic solvent types [69]. Some other studies also demonstrated the feasibility of using acidic aqueous solvents for the electrospinning of gelatin [70,71].

Some organic solvents including trifluoroethanol (TFE), trifluoroacetic acid (TFA) and hexafluoro isopropanol (HFIP) are also widely explored to dissolve gelatin as spinning solutions for the generation of electrospun nanofibers. For example, TFE was demonstrated to be a good solvent of gelatin, and dissolving gelatin in TFE exhibited an excellent spinnability. The smallest fiber diameter was roughly 100 nm when the concentration of gelatin in TFE was 5% (*w/v*) [72]. Dias et al. utilized TFA as a solvent for gelatin electrospinning [73]. The smallest mean diameter of nanofibers fabricated from gelatin/TFA solution was 157 nm. Zhan et al. employed HFIP as a solvent to electrospun gelatin into nanofibers, and the smallest mean diameter of nanofibers manufactured from gelatin/HFIP solution was 301 nm [74].

Although electrospun nanofibers made of pure gelatin have been successfully produced based on numerous different spinning solvents, the gelatin macromolecules in electrospun nanofibers commonly possess random coil conformation, inevitable resulting in inferior water-resistant capacity and poor mechanical properties. The previous studies demonstrated that the electrospun nanofibers made of pure gelatin may dissolve immediately once contacted with water [75,76]. To address these issues, lots of different physical or chemical crosslinking strategies have been developed to process the as-fabricated electrospun gelatin nanofibers [77,78]. Physical crosslinking of electrospun gelatin nanofibers is mainly based on some physical forces including hydrogen bonds, polar bonds, and van der Waals force as well as electrostatic interaction. Wang et al. employed pullulan, which could form hydrogen bonds with gelatin, to stabilize the electrospun gelatin nanofibers, leading to obviously enhanced mechanical properties [79]. Gungor et al. used a thermally crosslinked strategy to stabilize the structure and improve the water-resistant property [80]. In addition, an ultraviolet (UV) irradiation was used to physically crosslink the electrospun gelatin nanofiber mats by Beishenaliev and coworkers [81]. The crosslinked nanofibers exhibited increased diameters, which were three times higher than un-crosslinked nanofibers. The crosslinking process also improved the water-resistant ability, and the physically crosslinked nanofiber mats could remain in the cell culture medium for 14 days.

Comparing with physical crosslinking, chemical crosslinking can impart the gelatin nanofibers with more stable crosslinking networks, which are formed between gelatin molecules and chemical crosslinkers. Zhang et al. used 1-ethyl-3-(dimethyl-aminopropyl) carbodiimide hydrochloride (EDC) and N-hydroxyl succinimide (NHS) to crosslink the electrospun gelatin nanofibers [82]. They found that the crosslinked gelatin nanofiber mats presented obviously enhanced mechanical properties compared to the uncrosslinked control (Young modulus: 156 ± 36 MPa vs. 9.50 ± 3.51 MPa; Ultimate strength: 2.44 ± 0.75 MPa vs. 1.11 ± 0.22 MPa). Moreover, the water absorption capacity of crosslinked gelatin nanofiber mats was found to be decreased with the increasing of EDC content, because the strong chemical reactions occurred between the hydrophilic amino groups and carboxylic acid groups of gelatin molecules. Zhang et al., chemically crosslinked the electrospun gelatin nanofibers with glutaraldehyde vapor, and found that the crosslinked nanofiber mats presented 10 times higher Young’s modulus and ultimate strength than the uncrosslinked nanofiber mats [83]. Moreover, some other chemical crosslinkers, such as toluene 2,4-diisocyanate (TDI) [84], 1,4-butanediol diglycidyl ether (BDDGE) [73], oxidized phenolic compounds [85], genipin [86], have been reported to stabilize the electrospun gelatin nanofibers.

### 4.2. Electrospinning of Gelatin Blending with Other Polymers

Blend electrospinning is a simple and feasible strategy to fabricate gelatin-based nanofibers, and mixing gelatin with other biopolymers can effectively address the demerits of gelatin alone. Table 1 summarizes some representative electrospun gelatin-based blend nanofiber mats and their morphology and mechanical properties. The commonly used synthesized polymers include poly(ϵ-caprolactone) (PCL), poly(lactic acid) (PLA), poly(l-lactic acid) (PLLA), polyglycolic acid (PGA), poly(lactic-co-glycolic acid) (PLGA), polyurethane (PU), poly([2-(methacryloyloxy)ethyl] trimethylammonium chloride) (PMETAC), nylon 6, poly(vinyl alcohol) (PVA), Poly(ω-pentadecalactone-co-ε-caprolactone) copolymer (PDL-CL), etc. The usually used natural polymers contain hyaluronic acid (HA), chitosan (CS), cellulose acetate (CA), zein, fibrinogen, etc. The introduction of different natural or synthesized polymers can effectively improve the water-resistant capacity, and/or mechanical properties, and/or biological performances of finally generated gelatin-based composite nanofiber mats. For example, Dhandayuthapani et al. fabricated electrospun CS/gelatin blended nanofiber mats and demonstrated that the ultimate strength of CS/gelatin blended nanofiber mats (37.91 ± 4.42 MPa) was notably higher compared to electrospun gelatin nanofiber mats (7.23 ± 1.15 MPa) [87]. Moreover, the addition of CS could significantly improve the antibacterial activity of blended nanofiber mats. Kim et al. demonstrated that the introduction of PU into gelatin could significantly decrease the fiber diameter and improve the wet mechanical properties of as-electrospun blended nanofiber mats. Specifically, the fiber diameter, wet ultimate strength, and wet Young’s modulus were 2104 ± 140 nm, 0.6 ± 0.0 MPa, and 1.4 ± 0.1 MPa for electrospun gelatin nanofiber mats, while 406 ± 41 nm, 5.6 ± 0.9 MPa, and 3.2 ± 0.5 MPa for electrospun PU/gelatin (70/30, *w*/*w*) blended nanofiber mats [88].

Except for the blending of gelatin and single biopolymers, some studies have employed two, or more than two, components to blend with gelatin to generate gelatin-based composite nanofibers by electrospinning. Goudarzi et al. incorporated acetylated cellulose nanofibers (ACNFs) into electrospun gelatin/PCL nanofibers and demonstrated that the ACNFs loaded gelatin/PCL nanofiber mats possessed obviously increased ultimate strength than gelatin/PCL nanofiber mats (4.3 ± 0.1 MPa vs. 2.5 ± 0.1 MPa) [113]. Moreover, the addition of ACNFs could notably promote the proliferation of mouse L929 fibroblast cells. Massoumi et al. firstly loaded copper or zinc ions into halloysite nanotubes (HNTs), which were further loaded into gelatin/sericin nanofibers by blend electrospinning [114]. The Cu^2+^ contained nanofiber mats exhibited faster bactericidal activity and reduced viability to fibroblast cells. In comparison, the Zn^2+^ encapsulated nanofiber mats significantly promoted the attachment, viability, and collagen secretion of fibroblasts while maintained a favorable antibacterial capacity, presenting promising candidates as antibacterial wound dressings. In Cai et al.’s research, Fe_3_O_4_ nanoparticles were encapsulated into electrospun gelatin/CS nanofibers [115]. The nanoparticle-loaded gelatin/CS nanofiber mats were demonstrated to exhibit 155% increase of Young’s modulus and 128% augment of ultimate strength from the gelatin/CS nanofiber mats. Moreover, the antibacterial performance was also notably enhanced due to the encapsulation of Fe_3_O_4_ nanoparticles. The existing studies also reported the design and development of electrospun ternary nanofiber mats with different multifunctional performances for wound dressing applications, such as ferric oxide/gelatin/glycerol nanofiber mats [116], copper oxide nanoparticle/gelatin/PCL nanofiber mats [117], cerium oxide nanoparticle/gelatin/PCL nanofiber mats [118], sliver nanoparticle/gelatin/PVA nanofiber mats [119], tellurium nanoparticle/gelatin/PCL nanofiber mats [120], and halloysite nanotube/gelatin/PCL nanofiber mats [121].

**Figure 4:**
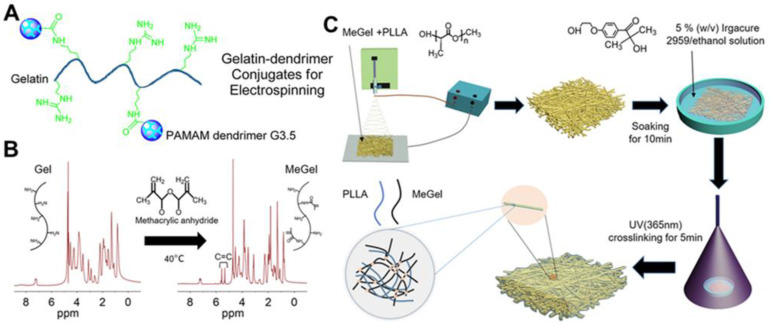
(**A**) Schematic illustration of the design of gelatin-PAMAM dendrimer G3.5 conjugates. (**B**) ^1^H-NMR of gelatin (Gel) and MeGel. The MeGel was synthesized by the methacryloyl substitution of Gel. (**C**) Schematic illustration of the construction of crosslinked MeGel/PLLA electrospun nanofiber mats by combining blend electrospinning with subsequent UV crosslinking process. (**A**) Reprinted with permission from ref [122], ACS Publications, 2013. (**B**,**C**) Reprinted with permission from ref [76], MDPI, 2022.

Although electrospun gelatin-based composite nanofibers have obviously enhanced performances compared to electrospun pure gelatin nanofibers, subsequent crosslinking strategies can also be used to stabilize the gelatin component and further enhance their properties of various composite nanofibers. An EDC/NHS system was utilized to covalently conjugate polyamidoamine (PAMAM) dendrimer G3.5 with a star-branched structure to gelatin molecules, and the modified gelatin was blend with unmodified gelatin for the fabrication of electrospun nanofibers in Dongargaonkar et al.’s study (Figure 4A) [122]. Subsequently, a photoreactive polyethylene glycol diacrylate (PEGDA) was employed to crosslink the modified gelatin component of as-electrospun nanofibers under an ultraviolet (UV) irradiation. The final generated nanofiber mats had increased structural stability and great water uptaking capacity. Sun et al. synthesized methacrylated gelatin (MeGel) through a methacryloyl substitution of gelatin (Figure 4B) [76]. The synthesized MeGel was blended with PLLA for electrospinning, and a subsequent UV crosslinking process was used to stabilize the MeGel component of MeGel/PLLA nanofiber mats (Figure 4C). They found that the UV crosslinking process dramatically improved the mechanical properties of MeGel/PLLA nanofiber mats and increasing the MeGel component notably decreased the mechanical properties but increased the water-absorbing capacity and biocompatibility to human dermal fibroblasts.

## 5. Electrospun Gelatin-Based Nanofiber Mats as Wound Dressings

The applications of electrospun gelatin-based nanofiber dressing materials for wound healing and skin regeneration have been extensively investigated during the past two decades. Electrospun gelatin-based nanofiber mats can effectively protect skin wounds against the invasion of external pathogens, allow great oxygen and moisture permeability, and provide good exudate-absorbing capacity [123,124]. Moreover, they also possess excellent biocompatibility and biodegradability, native ECM-like morphology and structure, as well as controlled physicochemical, mechanical, and biological properties, thus providing an appropriate healing-promoting microenvironment at the wound bed [125,126].

**Figure 5:**
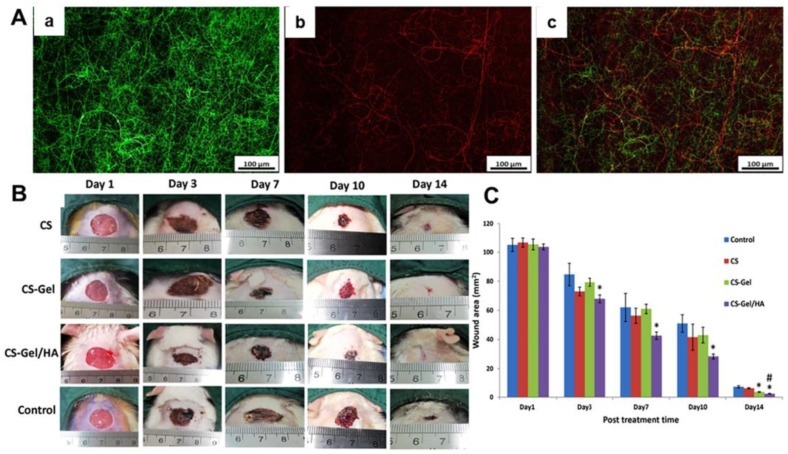
Design and development of composite wound dressings containing both CS-gelatin (Gel) nanofibers and CS-HA nanofibers. (**A**) Fluorescent images of (**a**) CS-Gel nanofiber mats with green color, (**b**) CS-HA nanofiber mats with red color and (**c**) merged CS-Gel/CS-HA nanofiber mats. (**B**) Photographs of wound-healing process using different dressings, i.e., CS nanofiber mats, CS-Gel nanofiber mats, CS-Gel/HA nanofiber mats, and medical gauzes (Control). (**C**) Statistical analysis of wound area of different experimental groups at predetermined time points. * *p* < 0.05 compared to control. # *p* < 0.05 compared to CS-Gel group. Reprinted with permission from ref. [129], Elsevier, 2020.

The electrospun composite nanofibers, by blending gelatin with some other components, have been widely designed to treat various skin wounds and have demonstrated significantly better healing outcomes compared with the traditional cotton gauzes. Ebrahimi-Hosseinzadeh et al. fabricated electrospun gelatin/HA nanofiber mats as wound dressing materials [127]. The in vivo results based on second degree burn wound showed that the gelatin/HA nanofiber dressings had a higher wound closure percentage (81.9%) than the commercial ChitoHeal gel wound dressings (65%), because the nanofiber dressings could effectively reduce the invasion of inflammatory cells and significantly promote the re-epithelialization. Electrospun nanohydroxyapatite-loaded gelatin/CA nanofiber dressings were manufactured by Samadian and coworkers [128]. The nanofiber dressings were demonstrated to obviously promote the in vivo neovascularization, collagen synthesis, and re-epithelialization, which exhibited a higher wound closure percentage (93.5 ± 1.6%) than the medical gauzes (51.23 ± 2.81%). Bazmandeh et al. employed a dual-electrospinning strategy to fabricate novel composite dressings containing both CS-gelatin (Gel) nanofibers and CS-HA nanofibers (Figure 5) [129] and found that the CS-Gel/CS-HA scaffolds exhibited an obviously higher cell proliferation (109%) than CS scaffolds and CS-Gel scaffolds after one day of culture. Moreover, the in vivo wound treatment results demonstrated that the wound closure using CS-Gel nanofiber dressings was obviously higher in comparison with the medical gauzes, and the CS-Gel/CS-HA composite nanofiber dressings showed the highest wound closure among all the experimental groups.

**Figure 6:**
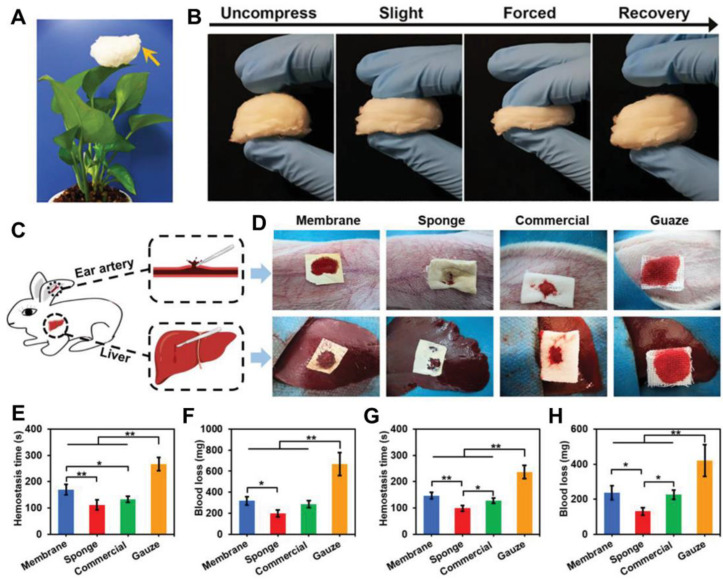
Design and development of a novel 3D electrospun gelatin nanofiber sponge as rapid hemostasis materials. (**A**) Photograph of the as-prepared 3D electrospun gelatin nanofiber sponge standing on a leaf. (**B**) Photographs of compress and recovery of gelatin nanofiber sponge with different degrees of external forces. (**C**) Schematic of an in vivo liver trauma model and an ear artery injury model using rabbit. (**D**) Photographs of hemostasis based on the two different injury models using four different samples. Membrane: electrospun 2D gelatin nanofiber mat; Sponge: 3D electrospun gelatin nanofiber sponge; Commercial: commercial 3D gelatin nonfibrous hemostatic sponge; Gauze: Commonly used medical gauze. (**E**) hemostasis time and (**F**) Blood loss of four different material groups based on the ear artery injury model. (**G**) hemostasis time and (**H**) Blood loss of four different material groups based on the liver trauma model. * *p* < 0.05, ** *p* < 0.01. Reprinted with permission from ref. [132], Wiley, 2021.

Modifying the structure and pattern of electrospun gelatin-based nanofiber dressings was also of significant importance to improving the healing efficiency of the finally obtained dressing materials. A bi-layered dressing constructed with one layer of electrospun gelatin/keratin nanofiber mat and one layer of commercial PU dressing by Yao and coworkers [130]. The in vivo wound study results indicated that the double-layered dressing significantly accelerated the wound healing process by promoting angiogenesis and re-epithelization after 14 days of acute wound treatment comparing with medical gauze and commercial wound dressing (Comfeel^®^, Peterborough, England). Similarly, Eskandarinia et al. also generated a double-layered dressing by electrospinning one layer of PCL/gelatin nanofiber mat on a commercial propolis extract-contained membrane [131]. The double layered dressing exhibited high antibacterial activity and biocompatibility, and notably better promoted the in vivo wound healing activities than commercial gauze. Xie et al. developed a 3D gelatin nanofiber sponge by using a combination of modified electrospinning technique and subsequent heat crosslinking treatment (Figure 6) [132]. The gelatin nanofiber sponge exhibited light weight, water-unsolvable, and high blood absorption ability, which could speed up the generation of platelet embolism and activate coagulation pathways. Both the in vivo liver trauma model and ear artery injury model demonstrated that the as-prepared 3D gelatin nanofiber sponge had superior higher hemostatic capacity comparing with the commercial 3D gelatin nonfibrous hemostatic sponge and medical gauze and showed a great potential for rapid hemostasis of large-scale acute wounds.

**Figure 7:**
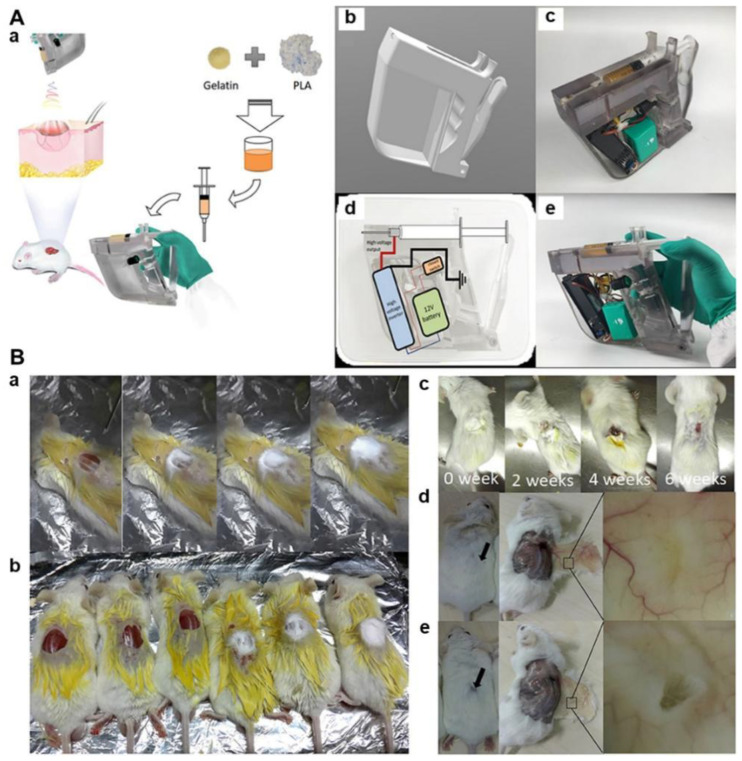
Design and development of in situ gelatin/PLA nanofiber wound dressings by using a 3D printed handheld electrospinning device. (**A**) Design and fabrication of 3D printed and handheld electrospinning device for gelatin/PLA blend electrospinning. (**a**) Schematic illustration of the application of in situ gelatin/PLA nanofiber dressing for wound covering and healing; (**b**) Computer-aided design of the electrospinning hand-holder; (**c**) Photograph of the 3D printed handheld electrospinning device; (**d**) Circuit design of the electrospinning setup; (**e**) Photograph of the finally generated handheld electrospinning setup. (**B**) In situ repair of the acute injury wounds using mouse models. (**a**) A series of photographs using in situ electrospun gelatin/PLA nanofibers to cover the wound; (**b**) Photographs of mouse wounds after covering or not; (**c**) A series of photographs of mouse wound after in situ treatment using electrospun gelatin/PLA nanofiber mat; (**d**) Photographs of regenerated skins after 8 weeks of treatment using electrospun gelatin/PLA nanofiber mat; (**e**) Photographs of regenerated skins after 8 weeks of treatment using commercial gauze. Reprinted with permission from ref. [133], Frontiers, 2021.

The advances of material science and engineering have inspired the design of in situ wound dressings. Compared with the conventional shelf-stored dressing products, the in situ formed dressings show several fantastic characteristics, i.e., simple and convenient operability, great flexibility and conformability without wrinkling on the wound site, and increased patient comfort. Moreover, the in situ dressings are especially suitable for wounds with irregular shapes. Most recently, Chen et al. fabricated a handheld electrospinning device by using a 3D printer, and the gelatin/PLA nanofiber mats were electrospun on the mouse wound site to realize the in situ wound repair by using the as-fabricated handheld electrospinning setup (Figure 7) [133]. The in vivo results indicated that the in situ electrospun gelatin/PLA nanofiber dressings exhibited an obviously increased repair for the acute injury wound comparing with the commercial gauzes.

## 6. Electrospun Gelatin-Based Nanofiber Mats Loaded with Bioactive Agents for Wound Healing Applications

Although the electrospun gelatin-based nanofiber mats have been widely demonstrated to promote the healing and regeneration of damaged skins to some extent, the functional recovery and regeneration efficiency are still unsatisfactory, especially for those large-scale acute wounds and various chronic wounds. The as-regenerated skin tissues commonly accompany abnormally reorganized scar tissues lacking necessary appendages like hair follicles, sebaceous glands, nerves, etc., which therefore cannot perform similar functions to intact skin. A combination of electrospun gelatin-based nanofibers with drug therapy seems to be a more appropriate strategy for various hard-healing wounds with severe complications. Table 2 summarizes some representative bioactive and therapeutics agents which are integrated with electrospun gelatin-based nanofiber mats for wound healing and skin regeneration applications. Various natural and synthesized components are used to provide some predetermined biological functions such as antimicrobial, anti-inflammation, oxidation resistance, hemostatic, and angiogenesis, as well as other healing-promoting capacities for shortening wound time and improving the functional recovery.

**Figure 8:**
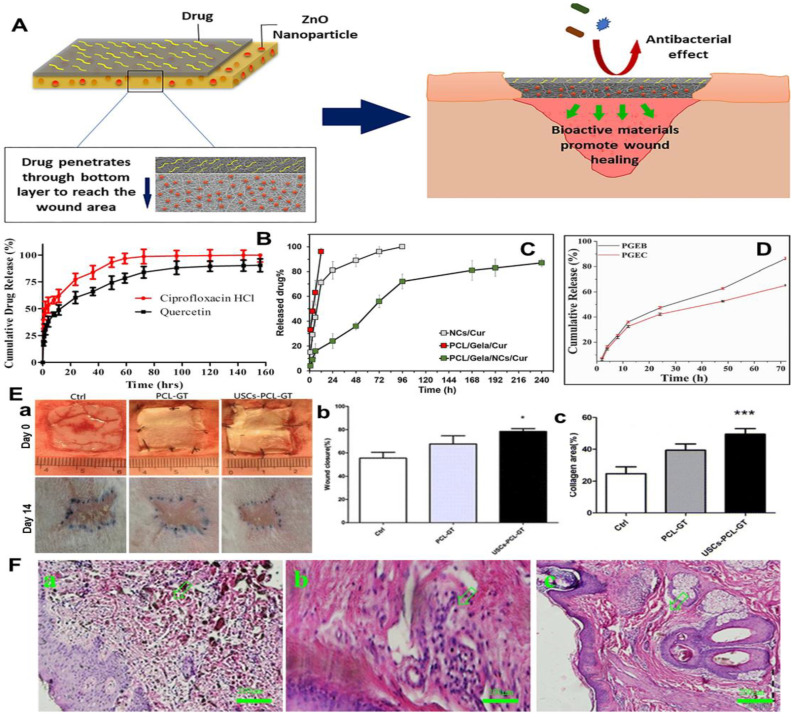
Design and development of dual-drugs loaded electrospun gelatin-based nanofiber mats for wound treatment. (**A**) Schematic illustration of the construction and working mechanisms of novel electrospinning double-layered PCL/gelatin nanofiber dressing. The above layer and bottom layer were made from amoxicillin loaded PCL/gelatin nanofibers and ZnO nanoparticles loaded PCL/gelatin nanofibers, respectively. (**B**) Cumulative release curves of quercetin and ciprofloxacin hydrochloride loaded electrospun PCL/gelatin nanofiber mats. (**C**) Cumulative release curves of EGCG from two different carriers. PGEB: EGCG directedly loaded PLCL/gelatin nanofiber mats; PGEC: EGCG-PLCL/gelatin core-sheath nanofiber mats. (**D**) Cumulative release curves of curcumin from three different carriers. NCs/Cur: curcumin loaded CS nanoparticles; PCL/Gela/Cur: curcumin loaded PCL/gelatin nanofibers: PCL/Gela/NCs/Cur: curcumin loaded CS nanoparticles were further encapsulated into PCL/gelatin nanofibers. (**E**) Regeneration of rabbit wound by using human urine-derived stem cells (hUSCs)-seeded electrospun PCL/gelatin nanofiber dressings. Ctrl: Untreated control group; PCL-GT: PCL/gelatin nanofiber dressing; USCs-PCL-GT: hUSCs-seeded PCL/gelatin nanofiber dressing. (**a**) Actual photographs of wound sites on day 0 and day 14. Statistical analysis for (**b**) wound closure ratio and (**c**) collagen area ratio on day 14. * *p* < 0.05, *** *p* < 0.01. (**F**) HE stained images from the wound sites treated with human placenta-derived mesenchymal stem cells (hPDMSCs)-carried electrospun gelatin nanofiber mats containing platelet-rich plasma (PRP). (**a**) Before treatment. (**b**) After two weeks of treatment. (**c**) After six weeks of treatment. (**A**) Reprinted with permission from ref. [165], Elsevier, 2020. (**B**) Reprinted with permission from ref. [166], Elsevier, 2019. (**C**) Reprinted with permission from ref. [168], Elsevier, 2022. (**D**) Reprinted with permission from ref. [169], Elsevier, 2020. (**E**) Reprinted with permission from ref. [170], Springer Nature, 2014. (**F**) Reprinted with permission from ref. [171], Elsevier, 2021.

Considering the complicated microenvironment at the wound bed, two or multiple bioactive and therapeutic components agents have been loaded into gelatin-based nanofiber mats during the electrospinning process. Jafari et al. fabricated a novel electropinning double-layered PCL/gelatin nanofiber dressing loading with ZnO nanoparticles and amoxicillin [165]. As shown in Figure 8A, one thin layer of amoxicillin loaded PCL/gelatin nanofibers was stacked on one thick layer of ZnO nanoparticles loaded PCL/gelatin nanofibers to construct the bi-layered dressing. The ZnO nanoparticles-encapsulated layer was designed to contact with the wound bed directly. The bi-layered dressing exhibited a sustained release of amoxicillin for 144 h in vitro after an initial burst release. Actually, the drug was expected to show a longer release time in vivo, because the layer loaded with ZnO nanoparticles would form a barrier that could hold back the drug diffusion into the wound bed. The double-layered dressing was demonstrated to significantly promote the in vivo angiogenesis and collagen deposition and effectively prevent scar formation, comparing with the commercial petroleum gauze. Ajmal et al. developed both quercetin and ciprofloxacin hydrochloride loaded PCL/gelatin nanofiber mats, which presented sustained release of both drugs after an initial rapid release (Figure 8B) [166]. The double drugs loaded PCL/gelatin nanofiber dressings confirmed great antibacterial and anti-inflammatory functions for accelerated wound healing in vivo. Similarly, both gentamicin sulfate and ciprofloxacin were loaded into alginate/gelatin nanofiber mats by Chen and coworkers [167]. The dual drug-loaded nanofiber dressing could effectively treat bacterial infection, and a complete wound closure of infected burn wound in rat was found after 21 days of treatment.

To reduce the early-stage burst release behavior of drug-loaded electrospun gelatin-based nanofibers, Li et al. fabricated core-sheath structured nanofibers by coaxial electrospinning. Specifically, the core layer was made from epigallocatechin-3-O-gallate (EGCG)/water solution, and the sheath layer was made from poly (L-Lactic-co-caprolactone) (PLCL)/gelatin dissolved in HFIP [168]. The in vitro drug release tests indicated that the EGCG release ratio from EGCG-PLCL/gelatin core-sheath nanofiber mats was 65% in 72 h, whereas it was 86% for the EGCG directly loaded PLCL/gelatin nanofiber mats (Figure 8C). The in vivo animal studies indicated that the EGCG-PLCL/gelatin core-sheath nanofiber dressings could significantly promote hemostasis and wound healing. Zahiri et al. firstly synthesized curcumin loaded CS nanoparticles, which were further encapsulated into PCL/gelatin nanofibers by electrospinning [169]. The in vitro drug release profiles shows that the accumulated release ratio of curcumin in the curcumin-CS nanoparticles/PCL/gelatin nanofibers was 23%, while it was 60% in the curcumin-CS nanoparticles in the first 6 h (Figure 8D). Moreover, the curcumin was completely released within 12 h and 106 h in the PCL/gelatin nanofibers directly loaded with curcumin and the curcumin-CS nanoparticles, respectively. In contrast, the curcumin-CS nanoparticles/PCL/gelatin nanofibers exhibited an obvious longer release time (roughly 240 h), which could provide anti-inflammatory, antioxidant, antibacterial performances for a long time, resulting in improved in vivo wound healing outcomes.

As for advanced and special drugs, living cells have also been employed to integrate with electrospun gelatin-based nanofiber mats for wound treatment applications. For example, Fu et al. demonstrated the human urine-derived stem cells (hUSCs) could secrete multiple growth factors including vascular endothelial growth factor (VEGF) and transforming growth factor-β1 (TGF-β1), and the hUSCs-seeded electrospun PCL/gelatin nanofiber dressings obviously accelerated wound closure in full-thickness rabbit skin wound models (Figure 8E) [170]. Meamar et al. found that the human placenta-derived mesenchymal stem cells (hPDMSCs)-carried electrospun gelatin nanofiber mats containing platelet-rich plasma (PRP) significantly promoted the skin regeneration of human diabetic foot ulcers by effectively reducing inflammation and promoting angiogenesis (Figure 8F) [171]. Lotfi et al. developed adipose tissue-derived mesenchymal stem cells (ADMSCs) and keratinocytesco co-seeded electrospun gelatin/chitosan/β-glycerol phosphate nanofiber dressings, which exhibited a significantly enhanced wound closure rate in the rat acute injury models [172].

## 7. Conclusions and Future Perspectives

During the past two decades, great endeavor has been devoted to blending polymer choice, solvent and functional additive selection, and post-treatment strategy like physical or chemical crosslinking, to improve the water resistance, control the degradation rate, and to increase the physical and biological performances of electrospun gelatin-based nanofiber mats. Moreover, considering the complexity of hard-healing wound sites, numerous different bioactive and therapeutic agents including natural drugs, synthesized drugs, growth factors, and other biomolecules, as well as living cells, have been incorporated with electrospun gelatin-based nanofibers by using some advanced electrospinning approaches like blend electrospinning, emulsion electrospinning and coaxial electrospinning. Despite all these advances made, there are still no commercial electrospun gelatin-based nanofiber wound dressings available in clinics. Firstly, the low productivity, poor reproducibility and a lack of standard operation methods and procedures of electrospun gelatin-based nanofibers severely holds back the realization of commercial translation. Secondly, although lots of existing studies have demonstrated that the integration of drug therapy and electrospun gelatin-based nanofiber mats are beneficial for the wound healing, what the best recipe component, concentration and release period are remains unknown, and the wound healing-promoting mechanisms of different bioactive components are also not clear. Thirdly, the as-reported improved wound healing efficiency by using drug electrospun gelatin-based nanofiber dressings are mainly achieved by rodent models which have different regenerative capacity and mechanisms with humans. We believe that our present review can provide reference and guidance for the future design and development of advanced electrospun gelatin-based dressing materials, and we can foresee the importance of electrospun gelatin-based strategies for clinical wound treatment applications in the future. Before that, it is necessary to improve the production and reproducibility, speed up the deep investigation of healing mechanisms, non-human primate tests and further clinical trials of electrospun gelatin-based wound dressings with or without therapeutic agents.

## Figures and Tables

**Table 1:** Lists of some representative polymers blending with gelatin for electrospinning.

Polymers Blending with Gelatin	Solvent	Mean Fiber Diameter (nm)	Young’s Modulus (MPa)	Ultimate Strength (Mpa)	Ref.
Fibrinogen	HFIP	133–309	Dry: 0.83–2.5 Wet: 0.003–0.46	Dry: 0.061–1.2 Wet: 0.013–0.009	[89]
Zein	70% (*v*/*v*) acetic acid	423–910	/	Dry: 0.2–6.3	[90]
Zein	80% (*v*/*v*) acetic acid	380–696	Dry: 72.1	/	[91]
Zein	HFIP	69–950	/	/	[92]
HA	Dimethyl Formamide (DMF)/water (0.5–2, *v*/*v*)	190–500	/	/	[93]
CS	TFA	90–279	/	/	[94]
CS	TFA/dichloromethane (DCM) (7/3, *v*/*v*)	180–196	/	Dry: 1.05–1.28	[95]
CS	TFA	120–220	/	Dry: 37.91	[87]
CA	HFIP	198–266	Dry: 47.92–95.44 Wet: 1.19–10.29	Dry: 1.59–3.06 Wet: 0.04–0.55	[96]
PDL-CL	HFIP	305	/	/	[97]
PCL	TFE	10–1000	Dry: 30.8	Dry: 1.29	[98]
PCL	TFE	200–800	/	/	[99]
PCL	TFE	300–600	/	/	[100]
PCL	chloroform/methanol (3/1, *v*/*v*)	291–1173	/	/	[101]
PCL	80% (*v*/*v*) acetic acid	444	/	/	[102]
PLA	HFIP	230–360	/	/	[103]
PLLA	HFIP	67–85	/	/	[104]
PLLA	HFIP	200–2100	Dry: 42.45–48.76	Dry: 2.45–3.48	[105]
PLLA	DCM/DMF (65/35 *v*/*v*)	500–560	Dry: 253–621	Dry: 6.0–12	[106]
PGA	HFIP	133–863	Dry: 32–72	Dry: 0.65–1.9	[107]
PLGA	TFE	479–774	Dry: 0.29–0.96	Dry: 1.44–3.59	[108]
PLGA	HFIP	500–1700	Dry: 81–101 Wet: 6–48	/ /	[109]
PU	HFIP	400–2100	Dry: 21.9–620.6 Wet: 2.4–3.2	Dry: 11.5–13.7 Wet: 2.0–5.6	[88]
PMETAC	formic acid/acetic acid (3/1, *v*/*v*)	429–2410	/	/	[110]
Nylon 6	formic acid and acetic acid (4/1, wt)	~10	/	/	[111]
PVA	Water	90–290	/	/	[112]

**Table 2:** Lists of some representative bioactive and therapeutic components introduced into electrospun gelatin-based nanofiber mats for wound treatment.

Materials	Bioactive Agent	Biological Performances	Animal Model	Ref.
Gelatin/PVA	Pine honey	Antioxidant	None	[134]
Gelatin/CS	Cinnamon	Antibacterial	None	[135]
Gelatin/PCL	Cinnamon	Antibacterial; Promoting wound healing	Acute injury wound with a square of 15 mm × 15 mm	[136]
Gelatin	Centella asiatica extract	Antibacterial; Promoting fibroblast proliferation and collagen synthesis; Accelerating wound healing	Acute injury wound with a square of 20 mm × 20 mm	[137]
Gelatin/Starch	Lawsonia Inermis (henna)	Antibacterial; Anti-inflammatory; Treating burn wound infections	Second-degree burn wound (A circle with a diameter of 5 mm)	[138]
Gelatin/PCL	Lawsone (2-hydroxy-1,4-naphthoquinone)	Antibacterial; Anti-inflammatory; Promoting wound healing	Acute injury wound with a circular area of 1.8 mm^2^	[139]
Gelatin/CA	Zataria multiflora	Antioxidant; Anti-inflammatory; Antibacterial; Accelerating wound healing	Second-degree burn wound with a square of 20 mm × 20 mm	[140]
Gelatin	Cinnamaldehyde (CEO), or Limonene (LEO), or Eugenol (EEO)	Radical scavenging; Antibacterial	None	[141]
Gelatin/PLGA	Hypericum capitatum var. capitatum (HCC) extract	Antibacterial	None	[142]
Gelatin/PCL	Clove essential oil	Antibacterial	None	[143]
Gelatin/PVA	Carica papaya	Antibacterial; Anti-inflammatory	None	[144]
Gelatin/PCL	Oregano oil	Antibacterial	None	[145]
Gelatin	Chondroitin sulfate	ECM mimicking; Promoting wound healing	Acute injury wound (a circular with a diameter of 15 mm)	[146]
Gelatin/PCL	Trimethoxysilylpropyl octadecyldimethyl ammonium chloride (QAS)	Cationic antibacterial agent; Broad-spectrum bactericidal	None	[147]
Gelatin/Silk fibroin (SF)	Astragaloside IV	Anti-scar; Accelerating wound healing	Second-degree burn wound	[148]
Gelatin/SF	Astragaloside IV	Anti-scar; Accelerating wound healing	Acute injury wound with a square of 15 mm × 15 mm	[149]
Gelatin/PCL	(+)-catechin	Antioxidant; scavenging reactive oxygen species (ROS)	None	[150]
Gelatin/PCL	Ketoprofen	Anti-inflammatory	None	[151]
Gelatin/PCL	Taurine (2-aminoethane sulfonic acid)	Non-essential sulfur-containing amino acid; Antioxidant; Promoting wound healing	Acute injury wound with a square of 15 mm × 15 mm	[152]
Gelatin/PLA	Ciprofloxacin	Broad-spectrum antibacterial; Treating infectious diseases	None	[153]
Gelatin/PVA	Gentamicin	Accelerating the wound healing; Reducing the treatment duration	Acute injury wound (a circular with a diameter of 8 mm)	[154]
Gelatin/PCL/ZIF-8	Gentamicin	Antibacterial; Accelerating the wound healing	Acute injury wound (a circular with a diameter of 20 mm)	[155]
MeGel/PCL	Cephalexin	Antibacterial; Improving re-epithelialization; Promoting collagen deposition	Second-degree burn wound (A circular with a diameter of 5 mm)	[156]
Gelatin/PVA	Cephradine	Broad-spectrum antibiotic (particularly against Gram-positive bacteria); Accelerating the wound healing process	Diabetic wound with a Ceph-resistant S. aureus infection (a circular with a diameter of 7 mm)	[157]
Gelatin/SF	Ceftazidime	Antibacterial; Preventing post-surgical adhesion	None	[158]
Gelatin/PLGA	Liraglutide	Promoting vascularization; Accelerating wound healing	Acute injury wound (a circular with a diameter of 20 mm)	[159]
Gelatin/PU	Silver-Sulfadiazine	Topical treatment for burn wound in clinics; Preventing burn infection; Promoting wound healing	Second-degree burn wound (A circular with a diameter of 15 mm)	[160]
Gelatin/Polyhydroxy butyric acid (PHB)	Silver-Sulfadiazine	Supporting enhanced re-epithelialization and MMP-9 production; Accelerating wound healing	Second-degree burn wound	[161]
Gelatin	Vitamins A and E	Antibacterial; Promoting the proliferation and collagen-specific gene expression of fibroblasts; Accelerating wound healing	Acute injury wound with a square of 30 mm × 30 mm	[162]
Gelatin/PLA	Epidermal growth factor (EGF)	Antibacterial; Anti-inflammatory; Promoting re-epithelialization; shortening healing time in venous ulcers	Second-degree burn wound (A circular with a diameter of 4 mm)	[163]
Heparin/Gelatin/PCL	Basic fibroblast growth factor (bFGF)	Promoting angiogenesis; Accelerating wound healing	Acute injury wound (a circular with a diameter of 10 mm)	[164]

## Data Availability

Not applicable.
